# A data-driven analysis, and its limitations, of the spatial flood archive of Flanders, Belgium to assess the impact of soil sealing on flood volume and extent

**DOI:** 10.1371/journal.pone.0239583

**Published:** 2020-10-01

**Authors:** Karen Gabriels, Patrick Willems, Jos Van Orshoven

**Affiliations:** 1 Department of Earth and Environmental Sciences, KU Leuven, Leuven, Belgium; 2 Department of Civil Engineering, KU Leuven, Leuven, Belgium; Imperial College London, UNITED KINGDOM

## Abstract

Soil sealing increases surface runoff in a watershed and decreases infiltration into the soil. Consequently, urbanization poses a significant challenge for watershed management to mitigate faster runoff accumulation downstream and associated floods. Hydrological models are often employed to assess the impact of land-use dynamics on flood events. Alternatively, data-driven approaches combining time series of land use geodatasets and georeferenced flooded zones also allow to assess the relationship between soil sealing and flood severity. This study presents such data-driven analysis using a spatially explicit archive of flooded areas dating back to 1988 in the Flanders region of Belgium, which is characterized by urban sprawl. This archived data, along with time series of rainfall and land use, were analyzed for three middle-sized river subbasins using two machine learning methods: boosted regression trees and support vector regression. The machine learning methods were found suitable for this type of analysis, since their flexibility allows for spatially explicit models with larger sample sizes. However, the relationship between soil sealing and flood volume and extent could not be conclusively confirmed by our models. This may be due to data limitations, such as the limited number of recorded historical floods, inaccuracies in recorded historical flood polygons and inconsistencies in the land use classifications. It is therefore stressed that continued consistent monitoring of floods and land use changes is required.

## Introduction

Land use changes impact the hydrology of a watershed. Especially soil sealing caused by urbanization can affect the hydrological processes of a watershed by decreasing infiltration and water storage in the soil, thus increasing rapid infiltration-excess overland flow and decreasing slow subsurface flow. Consequently, urbanization poses a significant challenge for sustainable land management as soil sealing leads to faster runoff accumulation downstream, which affects the occurrence and severity of flood events [1–3]. With urbanization increasing worldwide [4] and climatic conditions becoming more erratic [5], assessments of the hydrological impacts of land use changes are required in order to support future policy making regarding sustainable water resources management [6, 7]. The impact of land-use dynamics on flow regimes is often assessed using (semi-)distributed hydrological models incorporating land use information [2, 3, 8–10]. Based on these flow regimes, hydrodynamic models can then be employed to simulate flood extents [11–14]. However, flood inundation modelling, and the attribution of changes in flood regimes based on such models, has limitations due to uncertainties in model structure, model parameters and model inputs [15, 16]. Uncertainty in model structure arises from the type of model and its underlying assumptions [17, 18], while issues related to parameter calibration [19] and input data [12, 20] also add to the uncertainty of model results. By continuously monitoring observed flooded areas, time series of ever-increasing length are obtained. With the availability of these longer time series, the opportunity arises to assess the relationship between soil sealing and flood volumes and extents in a data-driven approach, taking into account the meteorological conditions and landscape configuration. These data-driven approaches relate the input variables directly to the observed outputs, and thus do not explicitly consider the underlying physical process. Given the complexity and nonlinearity of the hydrological processes involved, such as infiltration and evapotranspiration, the nonparametric and nonlinear data-driven methods are assumed suitable to assess the characteristics of flood phenomenon [21, 22].

Such empirical, data-driven analysis is tested in this study based on data for Flanders, a region of Belgium, which is prone to flooding: approximately 5% of the full territory has been flooded at least once in the period 1988–2016. The monitoring of these flood events from various sources lead to the creation of a geospatial flood archive with delineations of areas which have been flooded since 1988 [23, 24]. Furthermore, Flanders is characterized by urban sprawl resulting in a fragmented, complex landscape, referred to as a ‘rurban’ landscape [25]. These land-use dynamics together with the high population density make Flanders one of the most urbanized regions in Europe [26, 27]. Therefore, Flanders is an interesting study case to assess the impact of surface sealing on flood events using the boundaries of the flooded areas as recorded in the spatially explicit archive. Data from this archive, along with rainfall and land use data, were collected for three subbasins and analyzed using two Machine Learning (ML) methods: Support Vector Regression (SVR) and Boosted Regression Trees (BRT). A sensitivity analysis was performed to assess which factors impact the models most by alternately introducing variation into each of the factors.

## Materials and methods

### Geospatial data

#### Study areas

**Fig 1:**
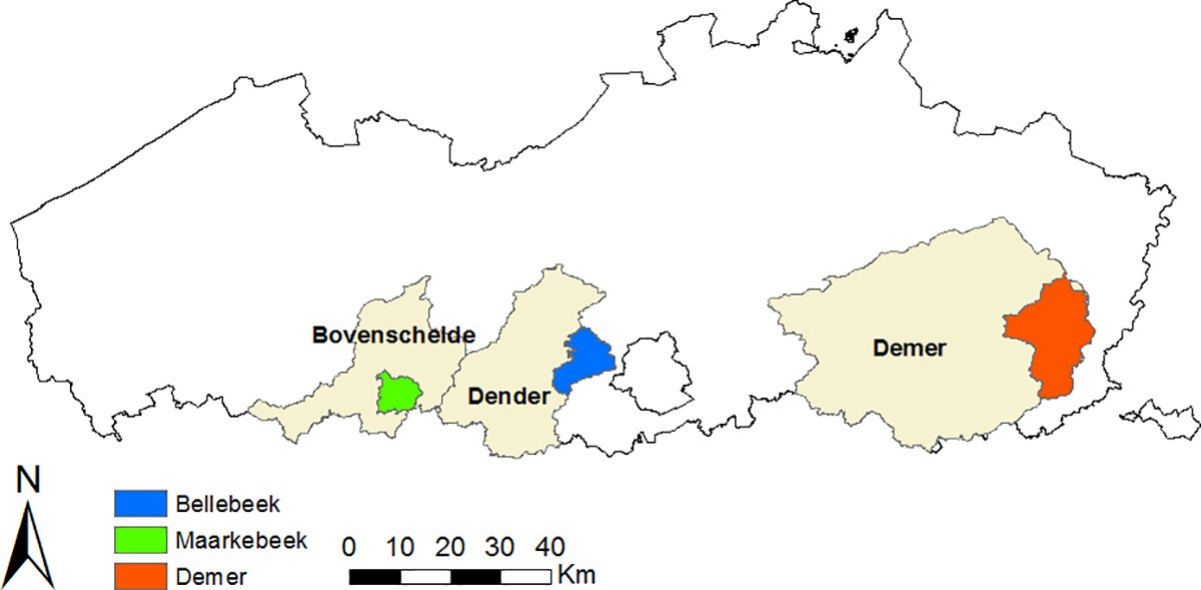
Location of the three study areas in Flanders, Belgium. Subbasins of the Maarkebeek (52 km^2^), Bellebeek (87 km^2^) and Demer (243 km^2^) [28, 29].

The study was carried out on three subbasins from three different primary river basins in Flanders, Belgium (Fig 1). The first study area, the Maarkebeek river basin, is situated in the Bovenschelde river basin and has an area of approximately 52 km^2^. The basin of the Bellebeek river (87 km^2^) is located in the Dender basin. Finally, an upstream subbasin of the Demer river was selected with an area of 243 km^2^.

#### Geospatial flood archive

**Fig 2:**
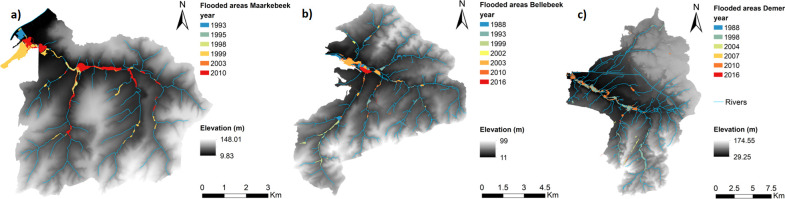
The spatial occurrence of floods between 1988 and 2016 in the study areas. (a) flood events in the Maarkebeek subbasin in six years, (b) seven flood events in the Bellebeek subbasin and (c) nine flood events in the Demer subbasin [23, 30].

The flood data were derived from the geospatial archive of flooded areas in Flanders [23]. This archive is published and maintained as a geodataset by the Flemish Environment Agency. Its latest update contains the contours of significant flooded areas in Flanders between 1988 and 2016 (Fig 2). The dataset is compiled from a variety of sources. Prior to 2000, when the archive was first assembled, flood contours were digitized from analogue maps recording flood extents. Later, mainly information provided by municipalities and aerial orthophotographs were used to update the archive. An updated version is released approximately every four years.

In the Maarkebeek subbasin, flood events were recorded in six years (1993, 1995, 1998, 1999, 2003 and 2010), while in the Bellebeek subbasin there were floods in seven years (1988, 1993, 1999, 2002, 2003, 2010 and 2016). Finally, in the Demer subbasin, nine flood events occurred: one event in 1988, 1998, 2004 and 2007, three in 2010 and two in 2016. These flood events were recorded in the spatial flood archive in respectively 48, 117 and 184 flood polygons in the Maarkebeek, Bellebeek and Demer subbasins. Table 1 provides the number of flood polygons for each flood event. The outlines of these flood polygons were combined in ArcMap 10.5.1 with a Digital Elevation Model (DEM) with a resolution of 5 m to derive the volume of water present in each of the flooded zones for the reported extent [30]. The flood volume (m^3^) and extent (m^2^) were assessed in the statistical analyses as dependent variables.

**Table 1:** Overview of flood events in the study areas, their meteorological characteristics and corresponding interpolated urban fractions.

SUBBASIN	Date	#	Vol (m^3^)	Area (ha)	PP (mm/hr)	PP (mm/6hr)	PS (mm/14d)	PS (mm/30d)	Urban (%)
MAARKEBEEK	30/12/1993	1	17,378	11.57	4.6	1.4	100	179	3.29
	25/01/1995	1	5,126	5.48	3.3	1.9	62	169	4.07
	09/09/1998	1	45,485	13.97	9.3	1.6	45	103	5.24
	25/12/1999	30	366,155	106.32	4.3	2	102	114	5.63
	01/01/2003	3	15,088	6.05	3.2	1.5	53	113	7.19
	13/11/2010	12	347,663	84.25	8.3	3.4	67	117	9.92
BELLEBEEK	12/03/1988	15	86,874	33.40	3.1	1	47	62	5.77
	19/12/1993	15	191,590	19.31	2.4	1.6	74	84	8.60
	24/12/1999	11	106,184	28.17	9.1	4	77	105	12.00
	05/08/2002	2	7,965	2.56	7.0	1.2	83	100	13.70
	01/01/2003	18	125,316	76.68	4.5	1.7	96	119	14.26
	12/11/2010	47	60,182	32.79	8.2	3.3	48	101	18.23
	01/07/2016	9	62,231	22.32	2.9	0.85	53	105	21.63
DEMER	12/03/1988	4	3,246	1.06	2.7	1.4	60	73	12.55
	13/09/1998	41	1,424,014	344.50	9.1	6.4	66	118	18.05
	6/08/2004	7	226,598	35.76	0.4	0.1	2	64	21.35
	8/11/2007	2	2,257	1.24	4.9	1.5	41	43	23.00
	14/08/2010	7	81,856	19.64	5.8	2.1	32	53	24.65
	12/11/2010	82	293,123	163.01	4.1	4.4	46	66	24.65
	13/12/2010	5	173,244	33.58	2.5	0.8	20	34	24.65
	26/05/2016	5	2,939	2.37	13.6	4.3	28	83	27.94
	01/06/2016	31	78,476	19.89	4.6	3.4	134	121	27.94

# = number of flood polygons associated with the flood event, Vol = volume of water in flooded zones, PP = peak precipitation during event, PS = accumulated precipitation prior to the flood event.

#### Meteorological data

Hourly precipitation data from the closest weather station of the Royal Meteorological Institute and the Flemish Environment Agency (www.waterinfo.be) were used to derive information on accumulated precipitation prior to the flood events and the intensity of the flood-inducing rainfall. Four derived variables were tested in the statistical analyses: the precipitation accumulation over the 14 days and 30 days prior to the flood event and the hourly and 6-hourly peak precipitation intensity 24 hours before the flood. An overview of this data for each flood event is provided in Table 1.

#### Land use data

The land use data were derived from three land use/land cover (LULC) maps valid for 1995, 2001 and 2012, which cover the area of Flanders. The 1995 land use map was derived from multispectral LANDSAT imagery using a maximum likelihood classification and describes the land use in Flanders in 27 classes, of which 21 occur in the study areas, with a resolution of 20 m [31, 32]. The land use map of 2001 [33] was derived from LANDSAT images using semi-automatic classification. It has a resolution of 15 m and distinguishes nine classes, with eight occurring in the subbasins, with a mean squared positional error of 18 m. The most recent land use map available is from 2012, which was constructed based on multispectral orthophotos and administrative parcel information using segment-based classification. It has a resolution of 5 m with 14 classes, all of which occur in the study areas, and a kappa-coefficient of 89.6%, which was derived by comparison with a sample of 1,252 points using an orthophoto of 2012 as reference data [34].

**Fig 3:**
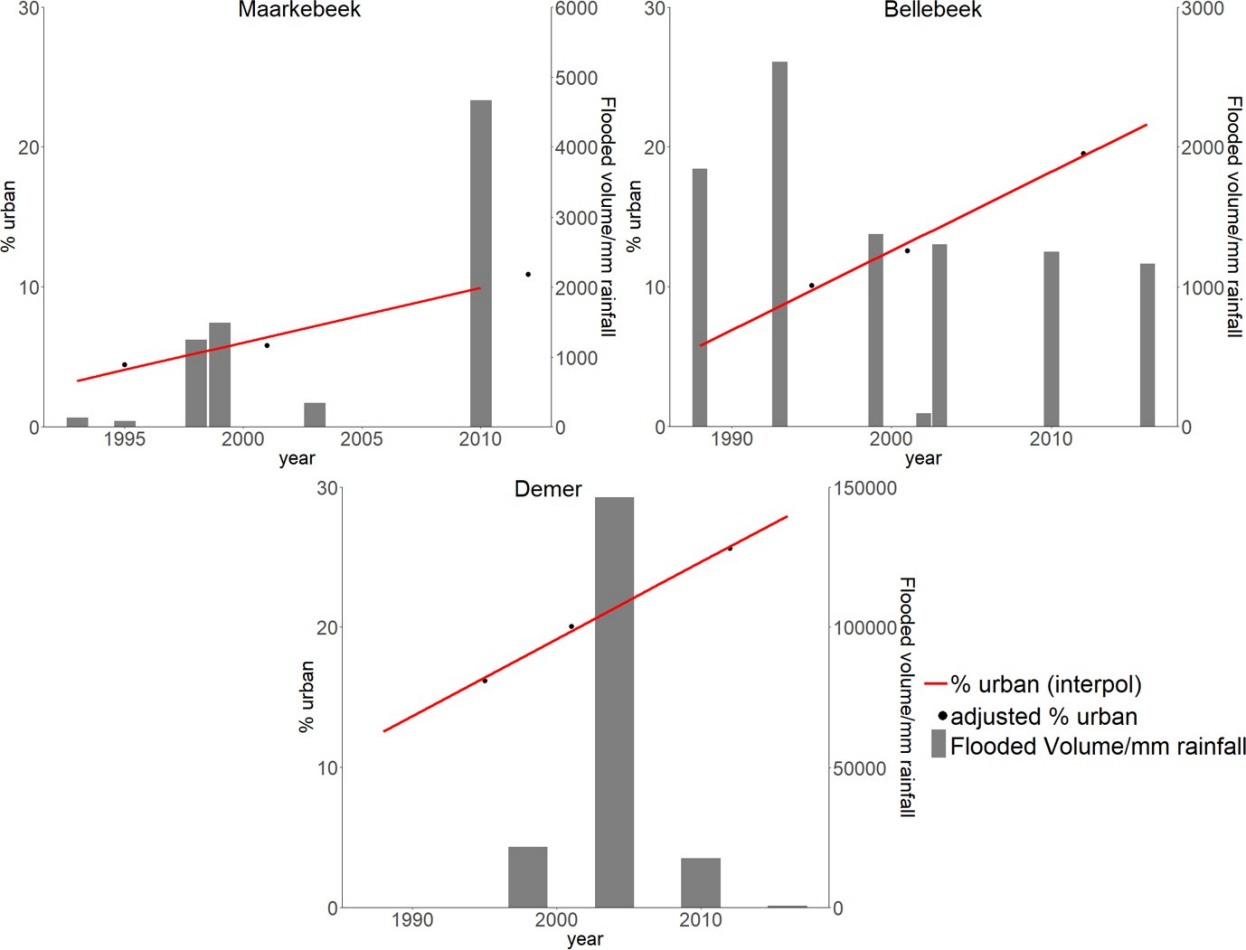
Percentage of urban area and flooded volume/mm rainfall. Corrected and interpolated percentages of urban areas and the flooded volumes (m^3^) divided by the accumulated precipitation 14 days prior to the flood events (mm) to provide the flooded volume per mm rainfall.

In order to geometrically and thematically align these land use maps, they were first resampled using the nearest neighbor algorithm to stack them at the resolution of 20 m. A land use change trajectory analysis was then applied to identify and correct improbable or impossible land use changes [35–38]. This analysis consisted in: (i) listing all LULC change combinations per pixel, (ii) expert-based evaluation of the likeliness of each combination and (iii) adjusting improbable changes when possible, e.g. changes from urban into another land use were reversed. This was done for every study area, after which the LULC maps were reclassified into the five classes of urban, arable land, forest, other green and water, with the urban land use class representing soil sealing. The percentages of adjusted urban area for the three land use maps are visualized in Fig 3, together with the volume of water in the flooded areas divided by the accumulated precipitation to obtain the flood volume per mm of rainfall. The Maarkebeek subbasin is the least urbanized, followed by the Bellebeek subbasin and the Demer subbasin. Urbanization takes place in all three study areas between 1995 and 2012, accelerating in the subbasins of the Maarkebeek and Bellebeek after 2001, and decelerating after 2001 in the Demer subbasin.

The machine learning methods incorporate the urban fraction upstream from the flood polygons as predictor: for flood events occurring before 1999, these fractions were derived from the adjusted 1995 LULC data, for flood events occurring between 1999 and 2005, the land use dataset of 2001 was used and for flood events after 2005, the land use dataset of 2012 was used to derive the urban fractions.

### Statistical methods

Machine learning aims to develop data-driven methods which improve with increasing experience or learning [39]. These data-driven learning methods provide a range of advantages, including a greater flexibility in data assumptions and less reliance on expert knowledge, which makes them also applicable in flood risk and susceptibility assessments [40–44]. A range of different methods have been developed, of which two were applied to our data: Support Vector Regression (SVR) and Boosted Regression Trees (BRT). The accuracy of each model was assessed with the Root Mean Square Error (RMSE) and relative RMSE, calculated by dividing the RMSE by the mean volume of water or area extent of the flooded polygons in each study area, i.e. 11,588 m^3^ and 47,424 m^2^ in the Maarkebeek subbasin, 5,473 m^3^ and 18,397 m^2^ in the Bellebeek subbasin and 12,423 m^3^ or 33,753 m^2^ in the Demer subbasin. The error estimates were calculated based on an independent test-set.

The observations used in the SVR and BRT are the individual flood polygons, since the absence of spatial correlation is not a prerequisite for these methods. This resulted in 48 observations in the Maarkebeek subbasin, 117 observations in the Bellebeek subbasin and 184 observations in the Demer subbasin. The response variables were the volume of water in these flooded polygons and their respective area. There were six predictors included in the models: accumulated precipitation, peak precipitation, upstream urban fraction, mean upstream urban area, edge density of the upstream urban area and the flood polygon’s flow accumulation. Two alternatives for the two meteorological predictors were tested, resulting in four possible combinations of peak precipitation (mm/hr; mm/6 hr) and accumulated precipitation (mm/14 days; mm/30 days). The urban fraction in the area upstream of the flood polygon was also included as a predictor, as were two indices of urban fragmentation and connectivity: (i) the mean area of an urban, upstream polygon (m^2^), and (ii) the edge density (m/m^2^) of the urban upstream area, defined as the total circumference of upstream urban areas divided by the total upstream urban area. A higher mean area indicates a more compacted urbanization, whereas a higher edge density indicates a more fragmented upstream urban area. The location of the individual flood polygons in the subbasin was represented in the model by the flow accumulation variable [45]. The flow accumulation was derived from the DEM and equals the number of upstream pixels that drain into an outlet. In this case, the most downstream pixel of each individual flood polygon was taken as the outlet. A pixel with a higher flow accumulation value is located more downstream in the subbasin.

#### Support vector regression

The Support Vector algorithm constitutes a supervised, nonlinear learning method which can be applied for classification or regression, in the latter case it is referred to as Support Vector Regression (SVR) [39, 44, 46]. In SVR, the inputs are first mapped to a hyperplane using a kernel function, which can be linear or radial. Next, a linear regression function is constructed in this hyperplane, which minimizes an ε-insensitive loss function. A regularization parameter controls the trade-off between model complexity and the loss function [44, 46, 47]. The models and their parameters were tuned and implemented using the ‘caret’ package in R-software, using repeated k-fold cross-validation [48], with ten repeats and five folds. The importance of the different predictors in the SVR was assessed using the feature selection procedure Recursive Feature Elimination (RFE), a well-known selection method for support vector algorithms. RFE first fits a model to all the predictors and ranks the features based on their importance in this model. Next, in an iterative process a model is trained while leaving out one feature based on its ranking, thus also determining the best feature subset size and composition [49]. The average adjusted R^2^ and its standard deviation over the different resampling loops were used to assess the importance of each predictor [48].

#### Boosted regression trees

Tree-based models, like BRT, use binary splits to partition the predictor space into homogeneous regions. These hierarchical decision trees automatically take into account interactions between predictors and are insensitive to outliers. However, small changes in training data can give different results [50]. Consequently, single tree models are unstable [51]. Boosting is a method to increase the model accuracy by combining a large number of single tree models to optimize the predictive performance. This is done in a stepwise, iterative way where a new tree is fitted on the residuals of the model [50]. Three parameters need to be specified to fit a BRT: (i) the learning rate, i.e. the weight given to each tree as it is added to the model, (ii) the tree complexity or the number of nodes in a tree and (iii) the number of trees required, which is controlled by the learning rate and tree complexity [46, 50]. The ‘train’ function in the ‘caret’ package of the R-software was used to set the optimal values of the learning rate and tree complexity for each of the study areas [48]. Next, the package ‘dismo’ of R was used to determine their optimal tree sizes and to develop the BRT [52]. Table 2 shows the results of this parameter tuning. The results of the BRT are visualized by partial dependence plots. These plots show the effect of a predictor on the response while accounting for the average effect of all other predictors. Though not a perfect representation, especially when the predictors are correlated, these plots provide a useful basis for the interpretation of the model [50].

**Table 2:** Results of the parameter tuning of the BRT for each of the study areas.

STUDY AREA	LEARNING RATE	TREE COMPLEXITY	NUMBER OF TREES
Maarkebeek	0.001	4	1450
Bellebeek	0.001	6	1600
Demer	0.001	4	1200

#### Sensitivity analysis

A sensitivity analysis was performed to assess which of the candidate predictors influence the flood volume models’ outputs most. In this analysis, the input values were perturbed One at A Time (OAT). The impact of these perturbations was assessed by a sensitivity index, where *M* is the number of factors in the model, *x*_*i*_ is the nominal value of the *i*-th input factor, *g* is the model’s response and *Δ*_*i*_ is the variation of the input factor [53, 54]:

$$S_{i}(x)=abs\left(\frac{g(x_{1},\ldots ,x_{i}+\Delta_{i},\ldots ,x_{M})-g(x_{1},\ldots ,x_{i},\ldots ,x_{M})}{\Delta_{i}}\right),$$ (1)

This OAT approach was performed for several *Δ*_*i*_ and the mean *S*_*i*_ and its standard deviation were calculated to assess each factor’s sensitivity: a higher S_i_ indicates a higher sensitivity [53]. The perturbations were chosen so that the entire range of the factor was covered. The perturbed nominal values were randomly based on one of the observations for each study area: the flood event recorded on 30/12/1993 was selected for the Maarkebeek subbasin, for the Demer subbasin the recorded flood event on 12/11/2010 was selected, and for the Bellebeek subbasin the observation on 05/08/2002 was selected.

## Results

### Support vector regression

Table 3 shows the RMSE and relative RMSE for the different SVR model configurations predicting flood volume and extent in the three studied subbasins. These error estimates show little variation when different combinations of meteorological predictors are implemented in the models. The errors are high for all three study areas, but lowest for the SVR models predicting flood extent in the Maarkebeek subbasin.

**Table 3:** RMSE and relative RMSE (%) of the support vector regressions for the three subbasins testing different meteorological predictors.

	Maarkebeek		Demer		Bellebeek	
**Vol**	**RMSE (m**^**3**^**)**	**rRMSE (%)**	**RMSE (m**^**3**^**)**	**rRMSE (%)**	**RMSE (m**^**3**^**)**	**rRMSE (%)**
PS14—PP	11,292	97	19,192	155	7,725	141
PS14—PP6	11,993	103	19,255	155	7,595	139
PS30—PP	11,449	99	19,329	156	7,899	144
PS30—PP6	13,219	114	19,404	156	7,928	145
**Area**	**RMSE (m**^**2**^**)**	**rRMSE (%)**	**RMSE (m**^**2**^**)**	**rRMSE (%)**	**RMSE (m**^**2**^**)**	**rRMSE (%)**
PS14—PP	27,722	58	67,064	199	39,688	216
PS14—PP6	27,756	59	64,360	191	39,006	212
PS30—PP	27,821	59	71,790	213	39,648	216
PS30—PP6	27,855	59	71,903	213	39,301	214

The dependent variable is the volume of water in an individual polygon (Vol; m^3^) and its area extent (Area; m^2^) (Maarkebeek: 48 obs., Bellebeek: 117 obs., Demer: 184 obs.). The independent, meteorological variables tested are accumulated precipitation (PS14; mm/14 days and PS30; mm/30 days) and peak precipitation (PP; mm/hr and PP6; mm/6 hr). Other independent variables included in the analyses are the flood polygons’ flow accumulation, fraction of the urban area upstream to each flood polygon, and mean area (m^2^) and edge density (m/m^2^) of upstream urban patches.

The results of the Recursive Feature Elimination, performed with an accumulated precipitation over 14 days and hourly peak precipitation, are given in Table 4. The predictors are ranked based on the mean R^2^: a higher mean R^2^ indicates a higher importance of the predictor in the SVR model. The standard deviation (SD) provides information on the variability of the mean R^2^. The flow accumulation is ranked first by the RFE in each SVR, the accumulated precipitation is ranked low for all SVR models. The fraction of upstream urban area is ranked third out of six in the SVR of the Maarkebeek subbasin, fourth in the Demer subbasin and fifth in the Bellebeek subbasin. The fragmentation indices, edge density and mean area of the upstream urban areas, are ranked low in the SVR of the Maarkebeek and higher in the SVR of the Demer and the Bellebeek.

**Table 4:** Results of the Recursive Feature Elimination (RFE) of the support vector regression.

	MAARKEBEEK			DEMER			BELLEBEEK		
	mean R^2^	SD	rank	Mean R^2^	SD	rank	Mean R^2^	SD	rank
Flow Acc	0.46	0.075	1	0.079	0.013	1	0.72	0.064	1
PeakP	0.31	0.063	2	0.042	0.015	5	0.022	0.013	4
PrecSum	0.20	0.051	5	0.006	0.005	6	0.004	0.018	6
Upstream Urban	0.31	0.101	3	0.032	0.008	4	0.022	0.021	5
Mean Area	0.28	0.066	4	0.055	0.017	2	0.036	0.038	2
Edge density	0.20	0.063	6	0.036	0.010	3	0.024	0.018	3

The dependent variable is the volume of water in an individual polygon (m^3^) (Maarkebeek: 48 obs., Bellebeek: 117 obs., Demer: 184 obs.). The independent variables are the accumulated precipitation (PrecSum; mm/14 days), peak precipitation (PeakP; mm/hr), flow accumulation (Flow Acc), fraction of the urban area upstream to each flood polygon (Upstream Urban), mean area of upstream urban patches (Mean Area; m^2^) and edge density of upstream urban patches (m/m^2^). The mean R^2^ and its standard deviation (SD) over the different resampling loops are provided as measures of importance for each predictor. The rank of each predictor, based on its mean R^2^, is also given.

### Boosted regression trees

Table 5 shows the RMSE and relative RMSE of the Boosted Regression Trees for the three subbasins. The RMSE of the BRT models are high for both the flood volume and the area extent, with the relatively lowest errors obtained in the Maarkebeek subbasin. The error estimates show little variation when different meteorological predictors are used in the models.

**Table 5:** RMSE and relative RMSE (rRMSE, %) of the boosted regression trees for the three study areas predicting flood volume (m^3^) and extent (m^2^) using different meteorological predictors.

	Maarkebeek		Demer		Bellebeek	
**VOL**	**RMSE (m**^**3**^**)**	**rRMSE (%)**	**RMSE (m**^**3**^**)**	**rRMSE (%)**	**RMSE (m**^**3**^**)**	**rRMSE (%)**
PS14—PP	18,410	159	46,095	371	15,343	280
PS14—PP6	18,599	161	46,257	372	15,419	282
PS30—PP	18,507	160	46,207	372	15,446	282
PS30—PP6	18,996	164	46,086	371	15,423	282
**Area**	**RMSE (m**^**2**^**)**	**rRMSE (%)**	**RMSE (m**^**2**^**)**	**rRMSE (%)**	**RMSE (m**^**2**^**)**	**rRMSE (%)**
PS14—PP	92,495	195	87,984	261	38,474	209
PS14—PP6	92,527	195	87,683	260	38,787	211
PS30—PP	92,545	195	88,088	261	38,608	210
PS30—PP6	92,964	196	87,917	260	38,634	210

The dependent variables are the volume of water in an individual polygon (VOL; m^3^) (Maarkebeek: 48 obs., Bellebeek: 117 obs., Demer: 184 obs.). The independent variables are the accumulated precipitation (PS14; mm/14 days, PS30; mm/30 days), peak precipitation (PP; mm/hr, PP6; mm/6 hr), flow accumulation, fraction of the urban area upstream to each flood polygon, mean area and edge density of upstream urban patches.

**Fig 4:**
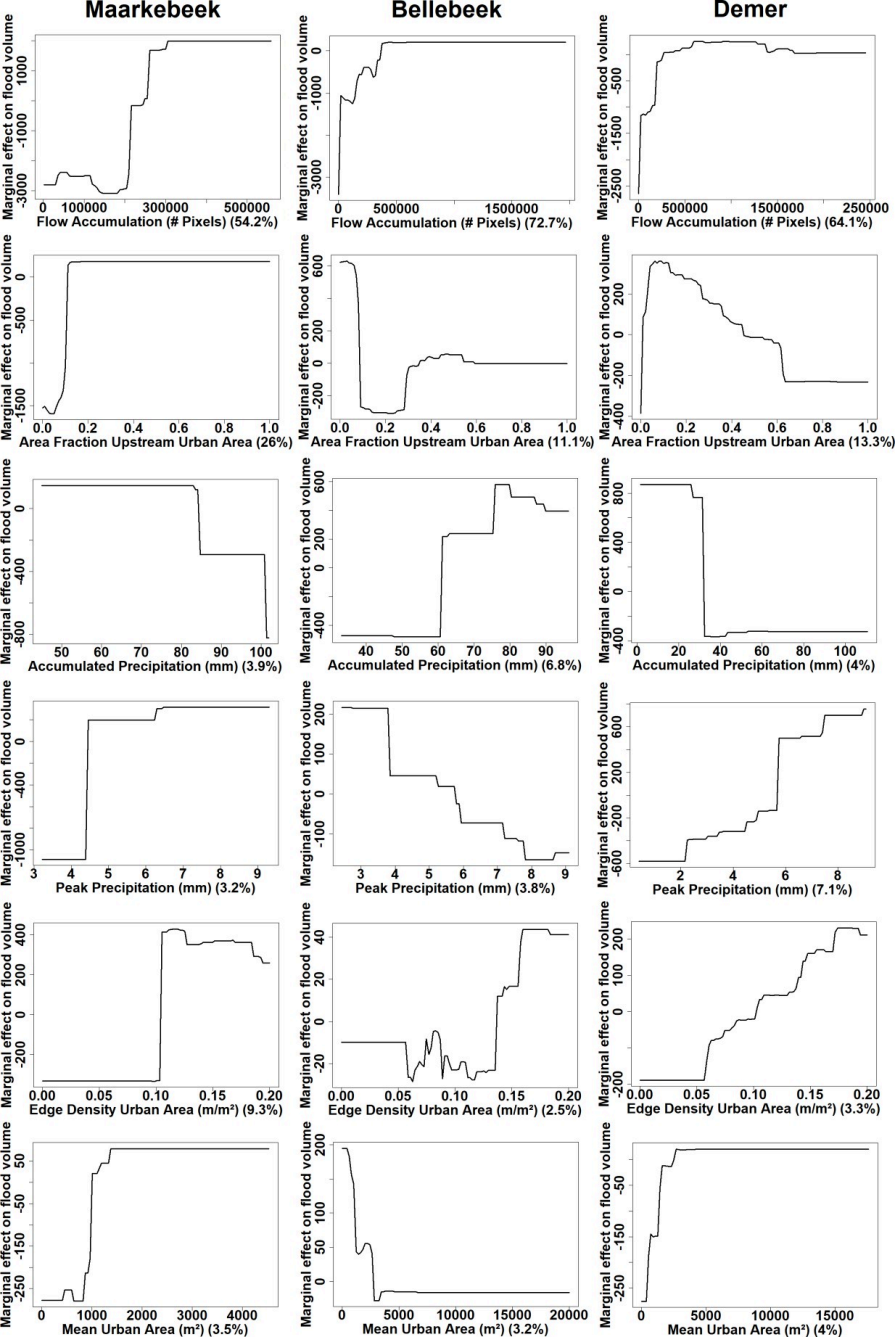
Partial dependence plots for the BRT models of the three subbasins of the Maarkebeek, Bellebeek and Demer. The importance of each predictor is given underneath the plots, expressed as a percentage.

Fig 4 shows the partial dependence plots for the Maarkebeek, Bellebeek and Demer subbasins for each predictor of the BRT models predicting flood volume, implementing 14 day accumulated precipitation and hourly peak precipitation as meteorological predictors. The flow accumulation is the most important predictor in the three BRT models with a relative importance of 54.2% in the Maarkebeek BRT model, 72.7% in the Bellebeek model and 64.1% in the Demer model. The fraction of urban area upstream of the flood polygons is the second most important predictor in the BRT models, with the highest importance in the Maarkebeek model (26%). The fragmentation indices, mean area and edge density of the upstream urban area, are of low importance (< 5%) in the BRT models, except for the edge density in the BRT model of the Maarkebeek basin (9.3%). The meteorological variables, accumulated precipitation and peak precipitation, are of relatively little importance in the models, which is also reflected by the results in Table 5 showing little variation with different meteorological predictors. These predictors are most important in the Demer model with an importance of 8.2% for the accumulated precipitation and 7.1% for the peak precipitation.

Overall, the partial dependence plots of flow accumulation in Fig 4 show that a higher value for flow accumulation results in a higher flood volume, indicating that zones close to the outlet are more prone to flooding. In the Maarkebeek model, the partial dependence plot indicates that higher upstream urban fractions contribute to flood volume, while this predictor has a negative effect in the Bellebeek and Demer models. Some of the partial dependence plots also indicate contra-intuitive and unlikely results. The partial dependence plot of the accumulated precipitation in the BRT model of the Maarkebeek basin indicates that a higher accumulated precipitation results in a lower volume of flood water. In the models of the Bellebeek basin and Demer subbasin, a higher peak precipitation or accumulated precipitation, resp., results in a lower flood volume.

### Sensitivity analysis

**Fig 5:**
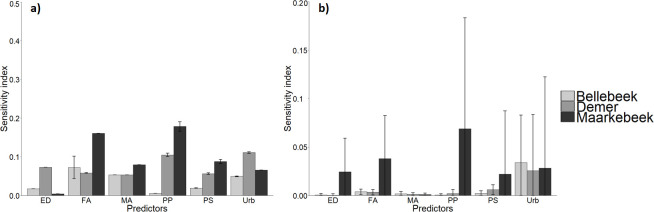
Results of the sensitivity analysis for the support vector regression models (a) and boosted regression trees (b). The sensitivity of the models for each factor is given: hourly precipitation peak (PP), 14 day accumulated precipitation (PS) and the area fraction of (upstream) urban areas (Urb), as well as edge density (ED), flow accumulation (FA) and mean urban area (MA).

The sensitivity analysis was performed on one single flood volume model configuration for each of the machine learning methods, namely the models implementing the 14 day accumulated precipitation and hourly peak precipitation, as the models implementing these meteorological factors have the lowest RMSE in predicting flood volume. The results of the sensitivity analysis are given in Fig 5. These results show that the SVR models are most sensitive to variations in the input data, although the BRT have the highest standard deviations of the mean S_i_. This is because the S_i_ of the BRT models follow the patterns shown in the partial dependence plots (see Fig 4): in some ranges of the factor values the S_i_ are zero, which means that a change in the factor does not result in a change of the flood volume; in other areas the sensitivity is higher, explaining the relatively high standard deviations of the S_i_. Overall, the models show a relatively high sensitivity to variations in the urban area fraction and the precipitation factors.

## Discussion

The relationship between soil sealing due to urbanization and flood volume and extent, derived from a spatial flood archive, was analyzed for three subbasins in Flanders using two machine learning methods, Support Vector Regressions and Boosted Regression Trees. Both these methods have been applied in environmental research [46, 55, 56], including flood susceptibility mapping and regional flood frequency analysis [40–44]. Machine learning methods do not assume a linear relationship between variables and allow for observations to be spatially correlated, making them also promising for a data-driven analysis of the relationship between urbanization and flood volume and extent, given the complexity of the relationship between floods and land use changes, including urbanization [1]. The individual flood polygons could therefore be implemented as observations in the SVR and BRT models and the locations of these polygons were included through their flow accumulation, thus increasing the sample size in each subbasin and making the statistical analyses spatially explicit. However, since this analysis derived the response variables from the spatial flood archive, the temporal dynamics of soil sealing on infiltration-excess surface runoff, resulting in higher and faster peak flows [1, 3], were disregarded.

The empirical analysis of Putro et al. [57] shows an upward trend in runoff totals in urbanized catchments. The hypothesis of a similar trend in flood volume and extent cannot be confirmed by our analysis using SVR and BRT, as no clear relationship with the urban area indicators is identified, model accuracy is low with both statistical methods and a number of unlikely associations, e.g. higher precipitation resulting in lower flood volumes, are present in all models. A possible explanation is the limited sample size, and consequently limited training set size, in our analyses, as only respectively 48, 117 and 184 flood events occurred between 1988 and 2016 in the Maarkebeek, Bellebeek and Demer subbasins. A regional analysis, pooling flood polygons from more subbasins than the three study areas included in this research, may improve model accuracy [58]. Overall, a lower RMSE was achieved with the SVR models, which were also found to be more sensitive to variations in the input data. The better model performance of the SVR methods is contrary to the findings of Heremans et al. [46], indicating that in sub-pixel land use classification, the accuracy of SVR models is more impacted by small training set sizes than BRT. The models with the lowest error were obtained for the Maarkebeek subbasin, which is the smallest, least urbanized study area. The urban fraction in the models of this subbasin also has a larger impact on flood volume compared to the other study areas (Table 4). This could be explained by the scale-effect [59]: the impact of land use and vegetation decreases with catchment size. This may indicate that the studied subbasins are too large to assess the effect of urbanization on flood volume and extent.

As in process-based hydrological and hydraulic models, uncertainty in the input data of the models is also an important source of error in data driven models [12, 20]. The meteorological, flood and land use data were therefore studied for potential errors.

The meteorological data, used to derive the meteorological predictors, were retrieved from the weather station closest to the studied subbasins. However, convective, local storm events causing floods may be underestimated by these point observations. This could cause inaccuracies when the precipitation station data are applied to local flood polygons. The precipitation indicators derived from these data showed a relatively high sensitivity in the models predicting flood volume, indicating that these inaccuracies may have a large impact. Integrating data from multiple weather stations or using spatially explicit rainfall maps derived from RADAR images may improve model results.

**Fig 6:**
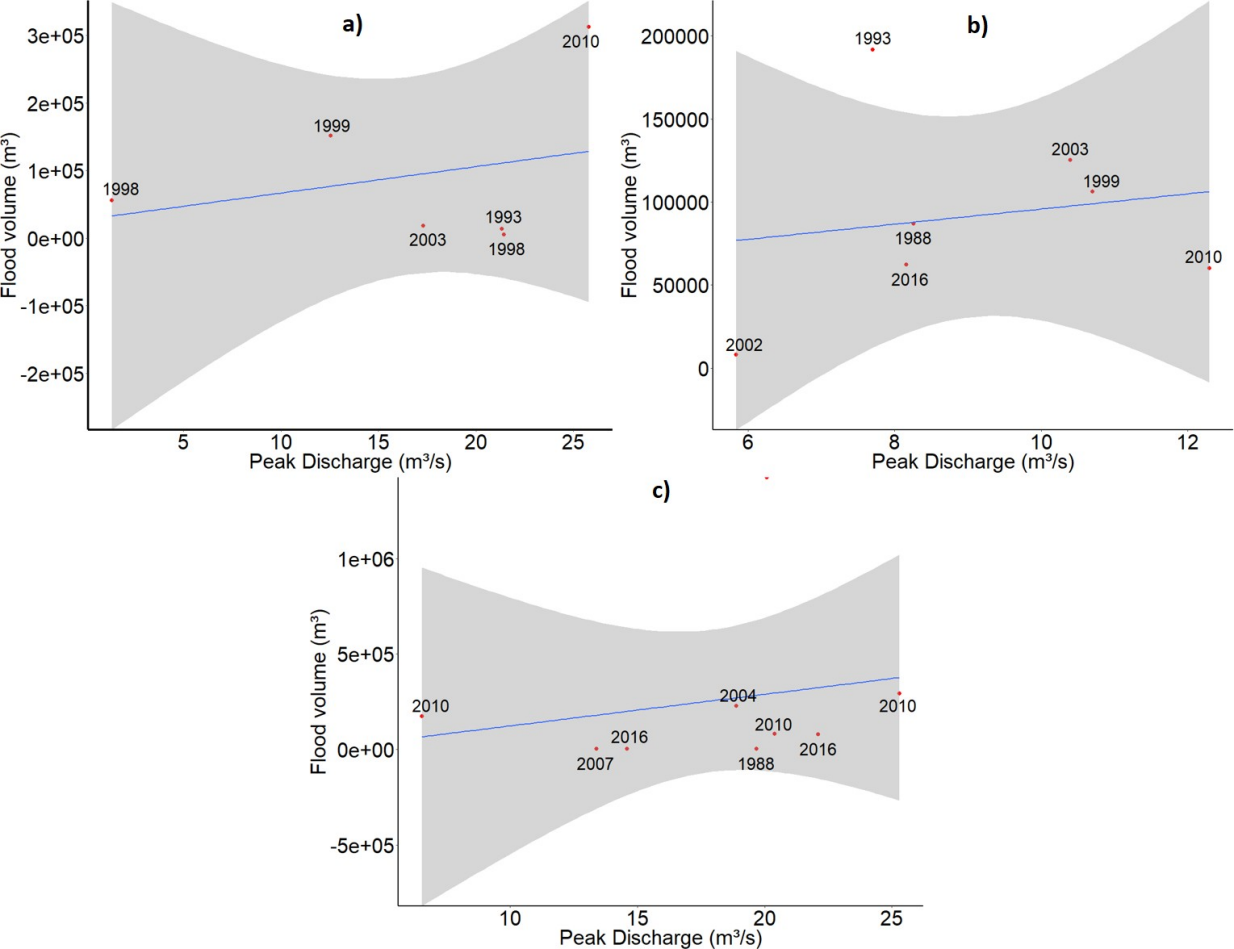
Relationship between the flood volume and measured peak discharge. Linear regression and confidence intervals of the volume of water in flooded areas (summed per event) versus the measured peak discharge during the flood events in the Maarkebeek basin (a; adj. R^2^ = 0.56, p-value = 0.055), Bellebeek (b; adj. R^2^ = -0.184, p-value = 0.803), Demer (c; adj. R^2^ = -0.095, p-value = 0.598).

The flood volumes and area extents, used as the dependent variable in the statistical analyses, were derived from the geospatial archive of the contours of the flooded areas in Flanders as recorded between 1988 and 2016. To assess the accuracy of the derived volumes, a linear regression was performed for the three study areas between the volume of water in flood polygons, summed per flood event, and the measured peak discharge during these flood events at the outlet of the basins [60]. It was assumed that a monotone increasing relationship exists between these variables: a higher peak discharge would result in a higher volume of water in the flood plains. The results of these regressions are shown in Fig 6. The best relationship was obtained in the Maarkebeek basin with an adjusted R^2^ of 0.56, the relationships in the Bellebeek and Demer basins resulted in negative adjusted R^2^. This exploratory analysis might indicate a poor relationship between the measured peak discharge and the derived flood volumes, which could indicate the presence of errors in the flood contours and DEM. Another possible explanation is that the recorded polygons do not always represent the maximum extent of the flooded zones, but an average or accidental extent. Especially flood contours recorded before the year 2000 may contain inaccuracies, since these polygons were digitized from analogue recordings. The consistent use of modern techniques, such as the use of drone technology or orthophotos to map the extent of flooded areas may help to reduce these errors.

The urban fraction is another important input factor in the statistical models, included in the machine learning methods as the fraction of upstream urban area from every flood polygon. These fractions were derived from three land use maps spanning the 1995–2010 period, a low number considering the rate of urbanization in Flanders [25]. The assumption was made that each land use map was representative for a number of years, linking several of the flood events to one land use dataset. These assumptions may have introduced errors in the estimated urban fractions, which can only be improved when more land use datasets become available. Another limitation for the land use datasets was the sparse or inadequate metadata, especially about the datasets’ quality. The metadata was largely missing for the 1995 land use dataset, while for the 2001 dataset only the mean squared positional error was reported without further explanation [33]. However, the metadata information regarding accuracy for the 2012 land use dataset was complete and indicated a high positional accuracy with a kappa-coefficient of 89.6% [34]. A land use trajectory analysis was performed to remove some of the inconsistencies in the classification between the land use datasets. However, some inconsistencies remain, especially between the land use map of 2001 and those of 1995 and 2012: the area fraction of forest and arable land are both lower in 2001 than they are in 1995 and 2012, indicating that this is most likely an inconsistency in this land use map and possibly due to the more generalized classes in 2001 (9 classes) as compared to 1995 (27 classes) and 2012 (14 classes). This difference in classification impedes the statistical analyses, as the sensitivity analysis indicated that the statistical models were sensitive to variations in urban fractions.

## Conclusion

The generally accepted hypothesis, that the expansion of soil sealing leads to increased flood volumes downstream, cannot conclusively be confirmed by the results of our analyses. Though the urban fraction is an important indicator in the machine learning models, the RMSE is high and the models reveal inconsistencies, such as a negative associations between accumulated precipitation and flood volume.

This finding may be partly explained by errors in the datasets: the boundaries present in the geospatial flood archive may not be fully correct due to digitization errors or may not always show the maximum extent of the floods; the land use data had different classification schemes, which could introduce errors in the derived urban fractions; and point observations from the meteorological stations may have missed local heavy precipitation intensities causing floods. The sensitivity analysis shows that the models, in particular the SVR models, are sensitive to these inaccuracies. Consistency in the monitoring of flood extents and in the classification of land use datasets is therefore important to allow data-driven analyses. Long-term flood monitoring will also help increase the currently limited sample size. Another possible explanation for the high RMSE and model inconsistencies could be the scale-effect [59], which states that the impact of land use and vegetation on flood events decreases with catchment size. This could be reflected in the fact that the models with the lowest error were obtained in the smallest study area, namely the Maarkebeek subbasin.

Despite these limitations, it was found that the machine learning methods applied in this study, Support Vector Regression and Boosted Regression Trees, were suitable for a data-driven analysis of the relationship between urbanization and flood volume and extent. Due to the more flexible data assumptions in these machine learning methods, the individual polygons could be considered as observations, thus increasing the observation sample size and allowing the location of these polygons to be included in the models. Consequently, the presented machine learning analyses are spatially explicit.

In conclusion, we can state that SVM and BRT are promising approaches for the empirical, data-driven assessment of the relationship between soil sealing and flood volume and extent. Clearly, there are data limitations to overcome, such as inconsistencies and inaccuracies as well as the limited length of the time series of flood extents. Of course, these limitations will also affect the performance of approaches based on mechanistic models. The data limitations indicate the need for a continued consistent monitoring of both flood events and land use changes in order to allow for more consistent outcomes of a data-driven analysis.

## Supporting information

S1 Data Dataset of the Maarkebeek subbasin. ID = flood polygon ID, year = year of the flood occurrence, Vol = volume of water in flood polygon (m^3^), Area = extent of flood polygon (m^2^), PrecSum14 = 14 day accumulated precipitation (mm/14 days), PrecSum30 = 30 day accumulated precipitation (mm/30 days), PP = hourly peak precipitation (mm/hr), PP6 = 6 hourly peak precipitation (mm/6 hr), Flow_Acc = flow accumulation in flood polygon (nr of pixels upstream of flood polygon), Urban = urban fraction upstream of flood polygon, Edgedens = edge density of urban area upstream of flood polygon (m/m^2^), Mean_Area = mean area (m^2^) of urban area upstream of flood polygon.
(TXT)

S2 Data Dataset of the Bellebeek subbasin. ID = flood polygon ID, year = year of the flood occurrence, Vol = volume of water in flood polygon (m^3^), Area = extent of flood polygon (m^2^), PrecSum14 = 14 day accumulated precipitation (mm/14 days), PrecSum30 = 30 day accumulated precipitation (mm/30 days), PP = hourly peak precipitation (mm/hr), PP6 = 6 hourly peak precipitation (mm/6 hr), Flow_Acc = flow accumulation in flood polygon (nr of pixels upstream of flood polygon), Urban = urban fraction upstream of flood polygon, Edgedens = edge density of urban area upstream of flood polygon (m/m^2^), Mean_Area = mean area (m^2^) of urban area upstream of flood polygon.
(TXT)

S3 Data Dataset of the Demer subbasin. ID = flood polygon ID, YMD = year, month and day of the flood occurrence, Vol = volume of water in flood polygon (m^3^), Area = extent of flood polygon (m^2^), PrecSum14 = 14 day accumulated precipitation (mm/14 days), PrecSum30 = 30 day accumulated precipitation (mm/30 days), PP = hourly peak precipitation (mm/hr), PP6 = 6 hourly peak precipitation (mm/6 hr), Flow_Acc = flow accumulation in flood polygon (nr of pixels upstream of flood polygon), Urban = urban fraction upstream of flood polygon, Edgedens = edge density of urban area upstream of flood polygon (m/m^2^), Mean_Area = mean area (m^2^) of urban area upstream of flood polygon.
(TXT)
